# Novel Antimicrobial Peptides EeCentrocins 1, 2 and EeStrongylocin 2 from the Edible Sea Urchin *Echinus esculentus* Have 6-Br-Trp Post-Translational Modifications

**DOI:** 10.1371/journal.pone.0151820

**Published:** 2016-03-23

**Authors:** Runar Gjerp Solstad, Chun Li, Johan Isaksson, Jostein Johansen, Johan Svenson, Klara Stensvåg, Tor Haug

**Affiliations:** 1 The Norwegian College of Fishery Science, Faculty of Biosciences, Fisheries and Economics, UiT The Arctic University of Norway, Tromsø, Norway; 2 Department of Chemistry, the Faculty of Science and Technology, UiT The Arctic University of Norway, Tromsø, Norway; nanyang technological university, SINGAPORE

## Abstract

The global problem of microbial resistance to antibiotics has resulted in an urgent need to develop new antimicrobial agents. Natural antimicrobial peptides are considered promising candidates for drug development. Echinoderms, which rely on innate immunity factors in the defence against harmful microorganisms, are sources of novel antimicrobial peptides. This study aimed to isolate and characterise antimicrobial peptides from the Edible sea urchin *Echinus esculentus*. Using bioassay-guided purification and cDNA cloning, three antimicrobial peptides were characterised from the haemocytes of the sea urchin; two heterodimeric peptides and a cysteine-rich peptide. The peptides were named EeCentrocin 1 and 2 and EeStrongylocin 2, respectively, due to their apparent homology to the published centrocins and strongylocins isolated from the green sea urchin *Strongylocentrotus droebachiensis*. The two centrocin-like peptides EeCentrocin 1 and 2 are intramolecularly connected via a disulphide bond to form a heterodimeric structure, containing a cationic heavy chain of 30 and 32 amino acids and a light chain of 13 amino acids. Additionally, the light chain of EeCentrocin 2 seems to be N-terminally blocked by a pyroglutamic acid residue. The heavy chains of EeCentrocins 1 and 2 were synthesised and shown to be responsible for the antimicrobial activity of the natural peptides. EeStrongylocin 2 contains 6 cysteines engaged in 3 disulphide bonds. A fourth peptide (Ee4635) was also discovered but not fully characterised. Using mass spectrometric and NMR analyses, EeCentrocins 1 and 2, EeStrongylocin 2 and Ee4635 were all shown to contain post-translationally brominated Trp residues in the 6 position of the indole ring.

## 1 Introduction

Increasing numbers of pathogenic bacterial strains are becoming resistant to antibiotics. More people in US hospitals now die from methicillin-resistant *Staphylococcus aureus* (MRSA) infections than HIV/AIDS and tuberculosis combined [1]. It is therefore a pressing need to find and develop antimicrobial agents as alternatives to classical antibiotics. Antimicrobial peptides (AMPs) are part of the immune system in both plants and animals and they are considered to constitute an evolutionarily ancient response to fight invading pathogenic microorganisms [2]. AMPs are evolutionary conserved and gene-encoded peptides, usually cationic, short (12–45) amino acid chains [3]. Most AMPs exhibit broad-spectrum activity towards both Gram-positive and Gram-negative bacteria. In contrast to commercial antibiotics where the development of resistance is a problem, bacterial resistance towards AMPs is much less pronounced [1, 4]. Because of their propensity to be rapidly metabolised in the gastrointestinal tract, peptides have been considered poor drug candidates. This problem has diminished somewhat in recent years with the development of new synthetic strategies to improve bioavailability and reduce metabolism of peptides, and bolstered by the development of alternative routes of administration [5, 6]. A large number of peptide-based drugs are now in clinical trials or being marketed, including AMPs [5, 7]. Today, more than 2600 peptides have been registered in the Antimicrobial Peptide Database [8], mainly from terrestrial sources. Marine invertebrates, although less studied, have proven to be a promising source for discovering AMPs with novel scaffolds [9].

Echinoderms are exposed to relatively high bacterial levels because they are often found in the photic zone where conditions for microbial growth are optimal. The survival of these organisms relies on the production of efficient antimicrobial components to defend themselves against microbial infections and fouling. As invertebrates in general [10], echinoderms do not have an adaptive immune system like the one recognised in vertebrates where specific memory towards pathogens is developed. Their innate defence system is mediated by the coelomocytes and compounds like complement factors, lectins, lysozymes and AMPs [11–13].

A number of AMPs have previously been found in echinoderms [13–20]. Examples include lysozymes that catalyse the hydrolysis of the peptidoglycans of the bacterial cell wall on Gram-positive bacteria and act as non-specific innate immunity molecules [17, 18]. A 6 kDa AMP in the coelomic fluid of the orange-footed sea cucumber, *Cucumaria frondosa*, was discovered but no sequence was reported [20]. Several antibacterial peptides from the coelomic fluid of the starfish *Asterias rubens* with masses around 2 kDa have also been discovered [19, 21]. Two of these peptides were identified as fragments of the histone H2A molecule, two peptides were identified as fragments of actin, and one was identified as a fragment of filamin A. A 5 kDa peptide having antistaphylococcal biofilm properties was discovered in the coelomocytes of the sea urchin *Paracentrotus lividus* [16]. The antibiofilm activity was suggested to be ascribed to beta-thymosin like fragments [22]. From the coelomocytes of the sea urchin *Strongylocentrotus droebachiensis*, two novel AMP families were characterised–the strongylocins [14] and the centrocins [23]. The strongylocins are cysteine rich peptides containing three disulphide bonds with MW in the 5.6–5.8 kDa range. Homologous genes have been discovered in *S*. *purpuratus* and their deduced peptide sequences named SpStrongylocins. Recombinantly produced SpStrongylocin analogues were also shown to be antibacterial [24]. The centrocins are a family of heterodimeric AMPs ranging between 4.4 and 4.5 kDa in mass. The peptides consist of two peptide chains: a 30 amino acid residue heavy chain (HC) and a 12 amino acid residue light chain (LC) connected by a single disulphide bond. Bioactivity studies have shown that the cationic HC is responsible for the antimicrobial activity of these peptides [23]. The HC of centrocin 1 displays potent activity against both bacteria and fungi and display antiinflammatory properties [25].

**Fig 1:**
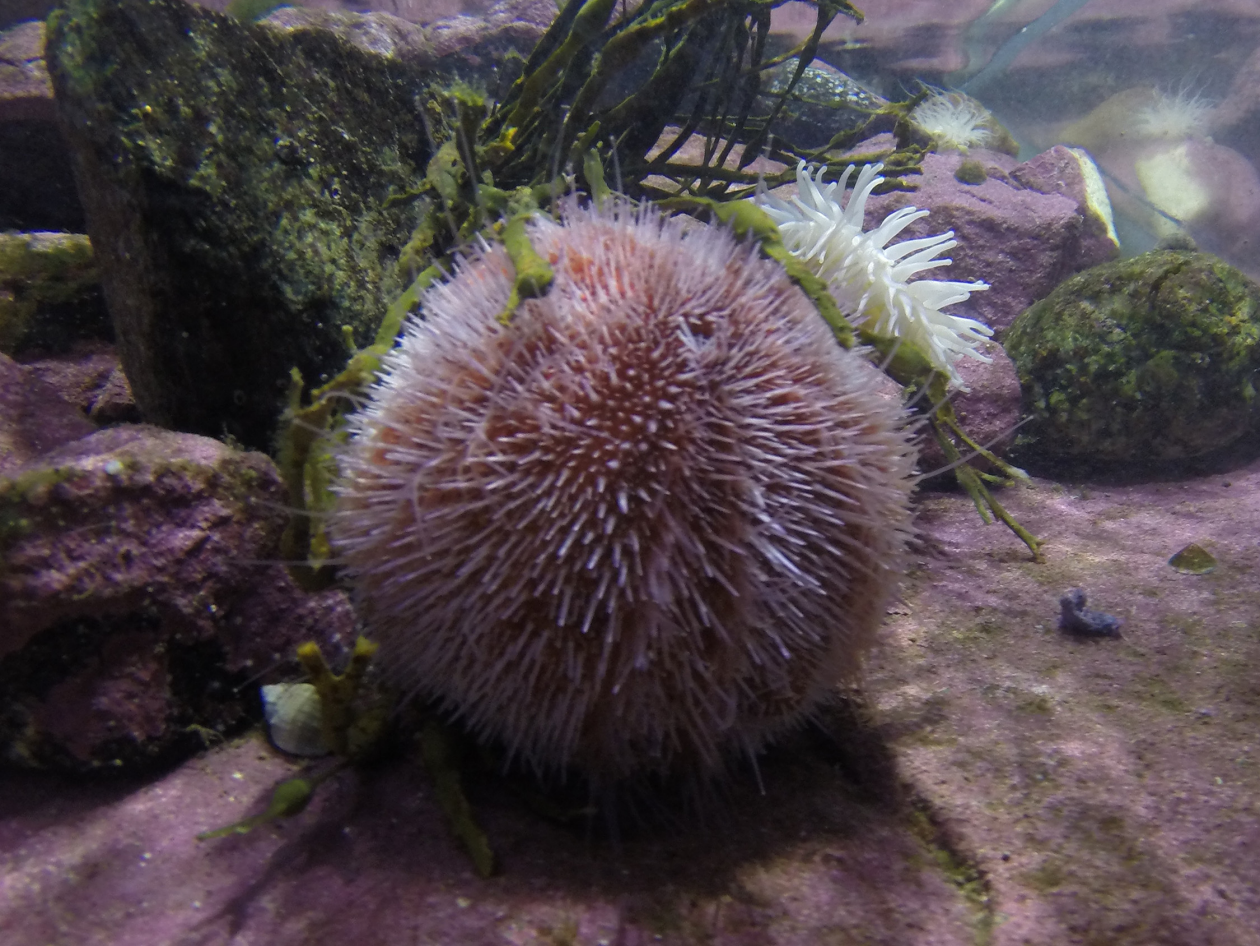
The Edible sea urchin, *E*. *esculentus*. Image credit: Runar Gjerp Solstad.

The Edible sea urchin, *Echinus esculentus* (Fig 1) has been reported to contain antimicrobial compounds [26], of which the quinone echinochrome-A has been identified [15, 27]. No AMPs have yet been discovered. The aim of the present study was to search for, isolate and characterise AMPs from the coelomocytes of the sea urchin *E*. *esculentus*. In this paper, we present the discovery of new AMPs belonging to the centrocin and strongylocin family of AMPs.

## 2 Materials and Methods

### 2.1 Ethics statement

All experiments performed in the present study were conducted in accordance with national and international guidelines and the ethical guidelines of UiT The Arctic University of Norway. For the haemolytic assay the collection of blood from a healthy donor was approved by the regional committee for medical research (REK 2014/1653).

In Norway, collecting wild *E*. *esculentus* for research purposes does not require specific permits. Our study does not involve endangered or protected species and sea urchins are not subject to any ethical animal use restrictions.

### 2.2 Animal collection and sample preparation

Live specimens of the Edible sea urchin, *Echinus esculentus* (Linnaeus, 1758) were collected twice: 1) autumn of 2012 (70 specimens) and 2) autumn of 2013 (71 specimens), by divers off the coast of Tromsø, Norway (70° 09.00**'** N 22° 15.22**'** E). The sea urchins were kept in continuously flowing, unaltered seawater at 4–8°C until sample collection.

Coelomic fluid was sampled from the animals 1–4 weeks after animal collection by penetrating the peristome with a scalpel and pouring the content into Ca^2+^/Mg^2+^ free anti-coagulating buffer [28] containing 70 mM EDTA and 50 mM imidazole in a 2:1 v/v ratio in 50 ml Falcon tubes (BD Biosciences, CA, USA) on ice. In total, approximately 5500 ml coelomic fluid was obtained. The mixture was subsequently centrifuged for 20 min at 4°C and 800 *g*. The pellet (coelomocytes) was collected by pouring off the cell-free supernatant and kept at -70°C until lyophilisation on a VirTis Genesis 35 EL freeze dryer (SP Industries, PA, USA) for 24 h. A total amount of 12.2 g (dry weight) of coelomocytes was sampled. All sample weighing was performed on a Sartorius Cubis MSA scale, Sartorius AG, Gottingen, Germany.

### 2.3 Extraction and purification

Lyophilised coelomocytes were extracted according to a previous protocol [29] with one modification: liquid-liquid extraction was carried out twice with 5% (w/v) lyophilised coelomocytes in 60% acetonitrile (ACN) containing 0.1% trifluoroacetic acid (TFA) (both from Sigma-Aldrich, MO, USA) for 24 h at 4°C. The combined extracts were partitioned into an aqueous phase (approximate total of 100 ml) at the bottom and an ACN-rich phase (approximate total of 150 ml) at the top by leaving it in a -20°C freezer for approximately 1 h. Both phases were dried in a ScanSpeed 40 vacuum centrifuge (Labogene ApS, Denmark) for 24 h. The ACN-rich phase was reconstituted in MQ-H_2_O (Millipore MA, USA) to a concentration of 10 mg/ml and subjected to antibacterial activity testing.

The aqueous phase (5.49 g) was reconstituted to 10 mg/ml (549 ml) in 0.05% TFA/H_2_O (v/v) and further subjected to solid phase extraction (SPE) on a reverse phase C_18_ 35cc Sep-Pak cartridge (Waters, MA, USA) according to [29] as follows. The extract was loaded onto the cartridge, previously conditioned with ACN, and equilibrated with 0.05% TFA/H_2_O (v/v). After washing of the loaded extract with 0.05% TFA/H_2_O (v/v), four stepwise elutions were performed with 10%, 40%, 80% and 100% ACN containing 0.05% TFA (v/v). The different fractions collected were dried under vacuum, reconstituted to 10 mg/ml and tested for antibacterial activity.

Due to its pronounced antibacterial activity (S1 Table), the 40% SPE eluate was further fractionated by reversed-phase high-performance liquid chromatography (RP-HPLC) using a preparative XBridge C_18_ (5 μm, 19 × 250 mm) column, a 717 autosampler, 600E pump system, 2996 photodiode array detector and an in-line degasser (Waters, MA, USA), all controlled by the Millennium 32, v4.00 software (Scientific Equipment Source, Ontario, Canada). The flow rate was set to 8 ml/min with an optimised HPLC protocol containing 0.05% TFA/H_2_O (v/v) and 0.05% TFA/ACN (v/v). The protocol started with 10 min of 0.05% TFA/H_2_O (v/v) followed by linear gradients increasing the 0.05% TFA/ACN (v/v) concentration from 0% to 18% over 8 minutes, 18% to 32% over 32 minutes, 32% to 50% over four minutes, and finally washing with 0.05% TFA/ACN (v/v) for five min and re-equilibration with 0.05% TFA/H_2_O (v/v) for five min. One minute (8 ml) fractions were collected automatically with a Gilson FC 204 fraction collector (Gilson, WI, USA), dried in a ScanSpeed 40 vacuum centrifuge for 24 h, and reconstituted in 500 μl MQ-H_2_O before antibacterial activity testing. Active fractions were analysed for purity, and bioactive peptides were detected using liquid chromatography UV-Vis mass spectrometry (LC-PDA-MS, see section 2.6). Impure, active fractions were fractionated again using the same experimental conditions but with fractions collected manually until pure peptides (estimated purity >90%) were obtained.

To perform extended bioactivity screening and structural analyses of individual peptides, more material of each peptide was needed. Multiple HPLC-runs, 23 injections in total, using the same conditions as described above, and by collecting fractions manually, where therefore performed. Pure peptide fractions (>90% as estimated with LC-MS) were pooled, lyophilised and weighed. Test solutions for bioactivity were prepared with MQ-H_2_O.

### 2.4 Microbial strains and antimicrobial activity assays

Organic phase extract, SPE eluates and HPLC-fractions were screened for antibacterial activity against two strains of Gram-positive and two strains of Gram-negative bacteria; *Corynebacterium glutamicum* (ATCC 13032), *Staphylococcus aureus* (ATCC 9144), *Pseudomonas aeruginosa* #1 (ATCC 27853) and *Escherichia coli* #1 (ATCC 25922). For extended antimicrobial bioactivity screening, the following bacterial strains were used, three strains of Gram-positive bacteria; *S*. *epidermidis* #1 (5179-R1) kindly provided by Holger Rohde [30], *S*. *epidermidis* #2, RP62A (ATCC 35984) and *Bacillus subtilis* (ATCC 23857) in addition to the Gram-negative *P*. *aeruginosa* #2, PA 01 (ATCC 15692, DSM 19880) and *E*. *coli* #2, NR 698 (MC4100/ptD4213).

Cultures stored at -80°C were smeared onto agar plates and cultured for 24 h at 35°C. One colony of each bacterial strain was transferred to 5 ml liquid Müller-Hinton (MH, Difco, Lawrence, KS, USA) medium in a glass tube and left shaking at room temperature overnight at 600 rpm. The cultures of actively growing bacteria (20 μl) were further inoculated in 5 ml MH medium and left shaking for 2 h at room temperature. The antibacterial assays were performed as previously described by [31]. Briefly, the bacterial cultures were diluted with medium to 1.3–1.5 × 10^4^ bacteria/ml final concentrations and an aliquot of 50 μl was added to each well in 96-well Nunclon^TM^ microtiter plates (Nagle Nunc Int., Denmark) preloaded with 50 μl test sample solutions, i.e. extracts, eluates or peptides.

The test plates were incubated for 24 h at 35°C with optical density (OD_595_) recorded every hour using an Envision 2103 multilabel reader, controlled by the Wallac Envision manager (PerkinElmer, CT, USA). Antibacterial activity was defined as a sample showing >90% inhibition (as measured by optical density) compared to the negative (growth) controls, consisting of bacteria and water. Oxytetracycline (20 μM) served as a positive (inhibition) control. The minimum inhibitory concentration (MIC) was defined as the lowest concentration of a sample displaying >90% inhibition.

The synthetic peptides (see section 2.11) were also screened for antifungal activity against *Candida albicans* (ATCC 10231), *Saccharomyces cerevisiae*, *Rhodotorula* sp., *Aureobasidium pullulans* and *Cladosporium sp*. The antifungal assay was performed as described previously [32]. Briefly, fungal spores were dissolved in potato dextrose broth (Difco, Lawrence, KS, USA) to a concentration of 4 × 10^5^ spores/ml. The spores (50 μl) were inoculated on 96-well Nunclon^TM^ microtiter plates containing the synthetic peptides (50 μl) dissolved in MQ-H_2_O. The fungal growth and MIC were determined visually after incubation for 24 h at room temperature. MIC was defined as the lowest concentration of peptide giving no visible fungal growth. The negative (growth) control contained medium and fungal solution.

ACN-phases and SPE eluates were tested with two-fold serial dilutions ranging from 5.0 to 0.005 mg/ml (5.0, 2.5, 1.25, 0.63, 0.31, 0.16, 0.08, 0.04, 0.02, 0.01, 0.005 mg/ml) whereas HPLC-fractions were tested undiluted for antibacterial activity. Pure, native peptides and synthetic peptides (described in section 2.11) were tested for antimicrobial activity in concentrations ranging from 100 to 0.1 μM in two-fold serial dilutions (100, 50, 25, 12.5, 6.25, 3.13, 1.56, 0.78, 0.39, 0.20, 0.10 μM). Antimicrobial assays involving natural peptides (i.e. fractionated natural material) were run only once due to limited quantities, whereas all other assays were run in triplicates.

### 2.5 Haemolytic activity assay

Synthesised peptide analogues of EeCentrocin 1 and 2 were screened for eukaryotic cell-toxicity with a haemolytic activity assay using human red blood cells as described previously [32]. The assay was performed on 96-well U-shaped microtiter plates (Nagle Nunc) with 50 μl peptide sample, 40 μl phosphate-buffered saline (PBS) and 10 μl red blood cells. The final peptide concentrations ranged from 100 to 0.1 μM in two-fold serial dilutions. After one hour of incubation at 37°C in a shaker, the plate was centrifuged at 200 *g* for 5 min and the supernatants (60 μl) were carefully transferred to a new flat-bottomed polycarbonate microtiter plate (Nagle Nunc) and absorbance at 550 nm was measured on a Synergy H1 multimode reader (BioTek, VT, USA). Cell suspension added 0.05% Triton X-100 (Sigma-Aldrich, MO, USA) in PBS served as positive (100% haemolysis) control and cell suspension added PBS served as negative (0% haemolysis) control. The percent haemolysis was calculated using the formula [(A_sample_-A_baseline_)/(A_triton_-A_baseline_)]×100. The experiment was performed in duplicates.

### 2.6 Two-dimensional liquid chromatography-mass spectrometry analyses

Antibacterial HPLC fractions and SPE eluates were analysed by liquid chromatography UV-Vis mass spectrometry (LC-PDA-MS) to identify antibacterial compounds and to perform purity determinations. The LC-PDA-MS system consisted of a 2695 separation module, a Sunfire C_18_ (5μm, 2.1 × 100 mm) column, a 2998 PDA detector reading from 190 to 500 nm in 1.2 nm increments, and a Micromass ZQ mass spectrometer controlled by Masslynx v4.1 software (all from Waters, MA, USA). Compounds were eluted by running a linear gradient of increasing ACN concentrations in water (both containing 0.05% TFA) from 5 to 50% over 16 min, using a flow rate of 0.2 ml/min. Samples (5–20 μl) were introduced to the MS and analysed in positive ESI mode. Ions were recorded in full scan mode in the 100–2000 *m/z*-range (See S2 Table for typical MS settings). The mobile phases were the same as when running HPLC (0.05% TFA/H_2_O (v/v) and 0.05% TFA/ACN (v/v)).

High-resolution mass spectrometry (HR-MS) was performed on a Thermo LTQ Orbitrap XL with an electrospray ion source (ION-MAX) coupled to an Accela HPLC-system (Thermo Fisher Scientific, MO, USA). A Supelco Ascentis Express (2.7 μm, 50 × 2.1 mm) C_18_ reverse phase column was used. The datasets were deposited to The Mass Spectrometry Interactive Virtual Environment repository (MassIVE, http://massive.ucsd.edu/ProteoSAFe/static/massive.jsp) database with accession number MSV000079515.

### 2.7 Peptide sequencing

Enzyme digestion with endoproteinase Arg-C and Edman degradation were performed by Eurosequence BV (Groningen, The Netherlands). Further sequence confirmation and elucidation of modified amino acids was achieved through trypsin digestion or reduction/alkylation and successive HR-MS. The protocol of Promega (Promega, WI, USA, available from http://no.promega.com/resources/protocols/product-information-sheets/n/sequencing-grade-modified-trypsin-frozen-protocol/) was followed regarding protease treatment. Briefly, peptides (700 μg) were dissolved in 6 M Guanidine HCl, 50 mM Tris HCl (pH 8) and 4 mM Dithiothreitol (DTT, Sigma-Aldrich, MO, USA) in a reaction volume of 100 μl. The reaction mixture was heated at 95°C for 20 min, cooled down to room temperature and added 550 μl 50 mM NH_4_HCO_3_ (pH 7.8). Porcine trypsin (Promega, WI, USA) was added at a 33:1 ratio (peptide:trypsin, 700:21 μg, 15.000 u/mg) and incubated for 16 h at 37°C. High-resolution LC-MS of the digested peptide was performed as previously described in section 2.6.

Reduction and alkylation was performed by dissolving ~20 nmol peptide in 100 μl 0.5 M Tris HCl/1 mM EDTA/6M Guanidine HCl and adding 5 μl 2.2 M DTT (Sigma-Aldrich, MO, USA). The peptide solution was flushed with N_2_ to prevent oxidation and incubated for 16 h at 37°C. After incubation, 5 μl 4-vinylpyridine (Sigma-Aldrich, MO, USA) was added to the solution and incubated for 20 min at 37°C. The reaction was stopped using RP-SPE with a C_18_ cartridge as previously described in section 2.3. The alkylated peptides were eluted with 80% ACN/H_2_O (v/v) containing 0.05% TFA.

### 2.8 Characterisation of full length cDNA

Total RNA was isolated from the pooled coelomocytes of three animals using the QIAZol^TM^ reagent in accordance with the manufacturer’s instructions (QIAGEN, MD, USA). Reverse transcription polymerase chain reaction (RT-PCR) was carried out using a rapid amplification of cDNA ends (RACE) kit (Clontech, CA, USA). Total RNA (1 μg) was used as a template to synthesise 5’ Ready-to-Go cDNA or 3’ Ready-to-Go cDNA according to the manufacturer’s instructions.

In order to obtain partial cDNA sequence (3’ region), degenerate oligonucleotide primed PCR (DOP-PCR) was performed as previously described [14]. Briefly, 0.5 μg of template (3’ Ready-to-Go cDNA), 10 × Optimised DyNAzyme™ Buffer, 1 μM of the forward primer EeCen 1DF (for EeCentrocin 1), EeCen 2DF (for EeCentrocin 2) or EeStrong 2DR (for EeStrongylocin 2) and reverse primers nested universal primer (NUP), 0.2 mM dNTP, 0.4 units DyNAzyme™ II DNA polymerase (Finnzymes, Finland) and water were mixed to bring the reaction volume up to 25 μl. DOP-PCR was performed according to the following cycle: 94°C for 5 min, 35 cycles at 94°C for 30 sec, 55°C for 30 sec and 72°C for 2 min, followed by a final extension at 72°C for 10 min. The DOP-PCR products of 3’ region were cloned into pGEM®-T vector and sequenced using primer Sp6 and T7. The correct sequences were confirmed by comparison of deduced amino acid sequences with sequences obtained by Edman degradation. The 5’ region of gene was cloned by using gene specific primer EeCen 1R, EeCen 2R or EeStrong 2R from the 3’ region and the primer NUP. The full-length nucleotide sequence was deduced based on the overlapping sequence of the obtained RACE product with existing partial cDNA sequence. Furthermore, the full length coding sequences were verified by PCR amplification using the primers (EeCen 1 AF/AR for EeCentrocin 1, EeCen 2 AF/AR for EeCentrocin 2 or EeStrong 2 AF/AR for EeStrongylocin 2) situated at the extreme ends of the open reading frame. An overview of all primers is presented in S3 Table. The sequences were submitted to GenBank with accession numbers KR494262, KR494263 and KR494264.

### 2.9 NMR analysis

NMR studies were performed on an Agilent (Varian) iNova spectrometer (Agilent Technologies, CA, USA) operating at 599.934 MHz for ^1^H equipped with a 5mm inverse cryogenically enhanced HCN probe (2^nd^ generation). All peptides were dissolved in D_2_O or H_2_O:D_2_O 9:1 in 3 mm Shigemi tubes matched for D_2_O. All acquired spectra and experimental parameters are summarised in S4–S6 Tables and deposited in the Biological Magnetic Resonance Data Bank (BMRB, http://www.bmrb.wisc.edu/) with accession number 26743.

The acquired spectra were referenced on the residual solvent signal δ_H1_ = 4.79 PPM and δ_C13_ from _γH1:-γC13_ = 3.976813 (water-*d*_2_). Data processing and figures were made using the MestReNova v9.0.1 and NMRPipe v8.1 [33] software, and peptide assignment was made using CARA v1.8.4.2 [34].

For comparison, NMR-spectra were obtained for 4-bromo-L-Trp (Amatek Chemical Co., Ltd., Jiangsu, China), 5-bromo-DL-Trp (Sigma-Aldrich, MO, USA), 6-bromo-DL-Trp (Biosynth AG, Staad, Switzerland), and 7-bromo-L-Trp (Amatek).

### 2.10 Data analysis and interpretation

The potential presence of peptide homologues was examined using the BLAST search engine (http://blast.ncbi.nlm.nih.gov/Blast.cgi) [35] on the National Centre for Biotechnological Information (NCBI) homepage. The blastp and blastn algorithms were used, searching for non-redundant protein sequences. Additionally, the built-in BLAST search functionality of the LAMP database (http://biotechlab.fudan.edu.cn/database/lamp/) [36] was used. Predicted signal sequences were determined using the SignalP 4.1 server (http://www.cbs.dtu.dk/services/SignalP/) [37] with the default setting for D-cutoff values and no TM regions selected. The cDNA sequence and deduced amino acid sequence of sea urchins were analysed using the BLAST program and the ExPASy Translate tool (http://web.expasy.org/translate/) with the genetic code set to standard. Alignments using ClustalW [38] and phylogenetic trees using the Neighbour-joining method [39] with evolutionary distances computed using the Poisson correction method [40] were constructed by the Mega 6.06 software (http://www.megasoftware.net/) [41]. Mass spectrum predictions were performed with the ChemCalc online prediction software (http://www.chemcalc.org/) [42] using the peptides tool with resolution set to 0.001. Graphs were made using Graphpad Prism v 6.00 for Windows (Graphpad Software, CA, USA). Row means, SD and linear regression was performed by the software.

### 2.11 Peptide synthesis

The non-brominated heavy chains (HC) of EeCentrocins 1 and 2 and the light chain (LC) of EeCentrocin 1 were synthesised commercially (GenicBio Ltd., Shanghai, China), as were the brominated HC of EeCentrocin 1 (HC-diBr, Isca Biochemicals, Devon, UK). The synthetic peptides were subjected to antimicrobial activity screening and haemolytic testing as previously described in sections 2.4 and 2.5.

The fragment, GW_Br_W_Br_R, of EeCentrocin 1 was synthesised (Isca Biochemicals) to perform mass spectrometric comparisons with the trypsinated N-terminal fragment of EeCentrocin 1. The brominated peptides were produced with both Trp residues substituted in the 6 position (i.e. 6-D/L-Trp) of the indole ring.

## 3 Results and Discussion

### 3.1 Isolation of AMPs

**Fig 2:**
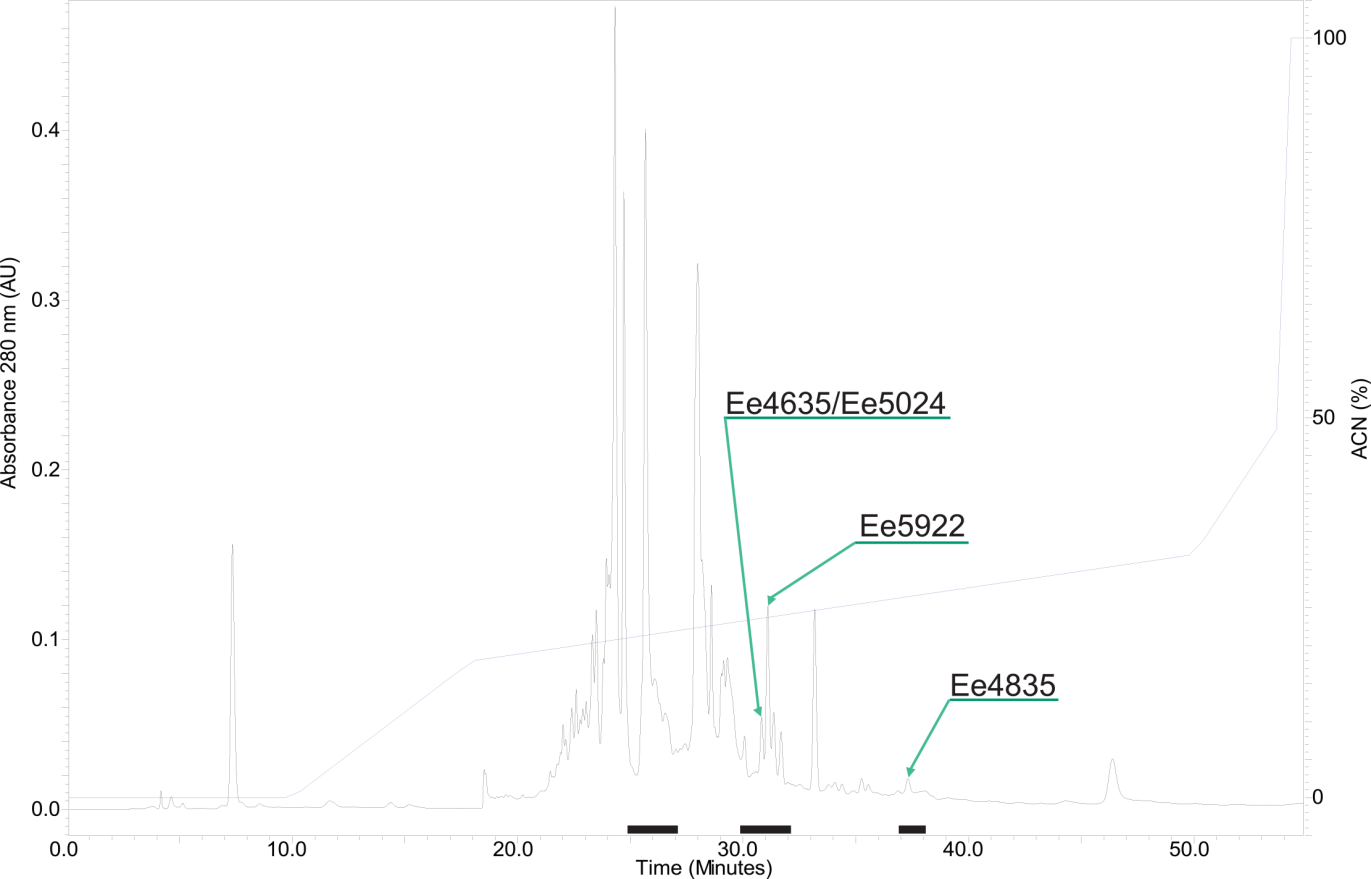
RP-HPLC chromatogram showing the separation of AMPs in the 40% SPE fraction of *E*. *esculentus* coelomocytes. The peptides were separated using a preparative C_18_ column using a flow rate of 8 ml/min, and an optimised HPLC gradient protocol of 0.05% TFA/ACN in 0.05% TFA/H_2_O for 60 min. One-minute fractions were collected and tested for antibacterial activity. Antibacterial fractions are marked with a black line under the chromatogram and peak fractions selected for further analysis are marked with arrows.

Antimicrobial compounds have previously been detected in the coelomic fluid and coelomocytes of various echinoderms [14–16, 20, 23, 43]. In the present study, four different SPE eluates obtained from an aqueous extract and one organic extract of *E*. *esculentus* coelomocytes were tested for antibacterial activity. Of the extracts, the 40% SPE eluate displayed the highest antibacterial activity (S1 Table). The antibacterial activity in this eluate ranged from 0.01 to 0.31 mg/ml, depending on test organism, and was therefore selected for further examinations. Out of the four bacterial strains tested (*C*. *glutamicum*, *S*. *aureus*, *P*. *aeruginosa* and *E*. *coli*), the Gram-positive *C*. *glutamicum* was the most sensitive overall. The 40% SPE eluate was fractionated by RP-HPLC and one-minute HPLC fractions were screened for activity against the same four bacterial strains. Growth-inhibiting properties were discovered in a series of fractions eluted with 20–30% ACN (Fig 2).

Three antibacterial fractions (eluting at 31, 32 and 38 min; Table 1) were further subjected to purification by RP-HPLC and manual fraction collection. Antibacterial activity was monitored on aliquots of the collected fractions during the purification process. Fraction 31 proved challenging to purify and after several repeated attempts to isolate the bioactive compound(s), only a semi-pure fraction was obtained. The fraction contained two peptides in an approximate 5:1 proportion with MW of 4635 and 5024 Da respectively (S1A Fig). Fraction 32 contained a 5922 Da peptide (S1B Fig) and fraction 38 contained a peptide with MW of approximately 4835 Da (S1C Fig). The purity of the peptides was monitored by absorbance at 220 nm and verified with LC-PDA-MS. The three peptide fractions were tentatively named Ee4635/Ee5024, Ee5922 and Ee4835 based on the MW of the most abundant peptides (See Fig 2 for chromatogram). Mass determination by HR-MS indicated the monoisotopic masses to be 4630.17, 5019.38, 5917.77, and 4830.20 Da, respectively (S2 Fig and Table 1).

**Table 1:** Peptide fractions with corresponding retention times (min) obtained by preparative RP-HPLC, monoisotopic masses (Da) of the native peptides obtained by HR-MS, and the partial amino acid sequences obtained by Edman degradation analysis. X denotes unidentified amino acids.

Peptide fraction	Retention time (min)	Mass (Da)	Amino acid sequence
Ee4635/Ee5024	31	4630.17/5019.38	Seq 1: XGGKTXGKIKHAARKGVGYASRGCRIAGH
			Seq 2: XGHKLRSSXNKVKHAVKKGAGYASGACRVLGH
Ee5922	32	5917.77	Seq 3: XNPFKKIAHRXCYPKNE
			Seq 4: RXACTVVAQ
Ee4835	38	4830.20	Seq 5: GXXRRTVDKVRNAGRKVAGFASKACGALGH
			Seq 6: DIGKYCGYAHALN

### 3.2 Primary structure elucidation

#### Edman degradation

An aliquot of each peptide fraction was reduced, alkylated and sequenced by Edman degradation. The analysis revealed multiple signals of amino acids of similar intensity in each position of the peptide fractions Ee4835 and Ee4635/Ee5024, suggesting that these fractions contained peptides composed of more than one peptide chain. The reduced and alkylated peptides were therefore subjected to RP-HPLC purification and successive Edman degradation of each chain. The analysis revealed that the peptide Ee4835 seems to have a heterodimeric structure, containing a heavy chain (HC) composed of 30 amino acids (Seq 5, Table 1) and a light chain (LC) composed of 13 amino acids (Seq 6, Table 1), possibly connected via a single disulphide bond. Additionally, the analysis exposed uncommon or modified residues (X) in positions two and three of the HC. Analysis of Ee4635/Ee5024 returned two sequences with similarity to the HC of Ee4835, probably belonging to the two different peptides known to be in the sample. The main peptide (Ee4635 giving rise to the largest signal intensity both in MS analysis and during sequencing) consisted of 29 amino acids (Seq 1, Table 1). Minor but distinct signals were also recorded for a 32 amino acid peptide (Seq 2, Table 1). Both peptide sequences contained a single cysteine residue and had uncommon or otherwise modified amino acids in different positions: 1 and 6 for Ee4635 and 1 and 9 for Ee5024. Although the HPLC chromatogram of the alkylated peptide fraction displayed additional peaks, no other sequences were obtained. This indicates that additional peptide fragments were present, but N-terminally blocked. Edman degradation analysis of Ee5922 revealed a partial 17 amino acid N-terminal sequence (Seq 3, Table 1). Enzymatic treatment with endoproteinase Arg-C and subsequent purification and sequencing of cleavage products revealed an additional 9 amino acid sequence (Seq 4, Table 1). The obtained amino acid sequences formed the basis for cDNA library construction.

#### Characterization of cDNA sequences

**Fig 3:**
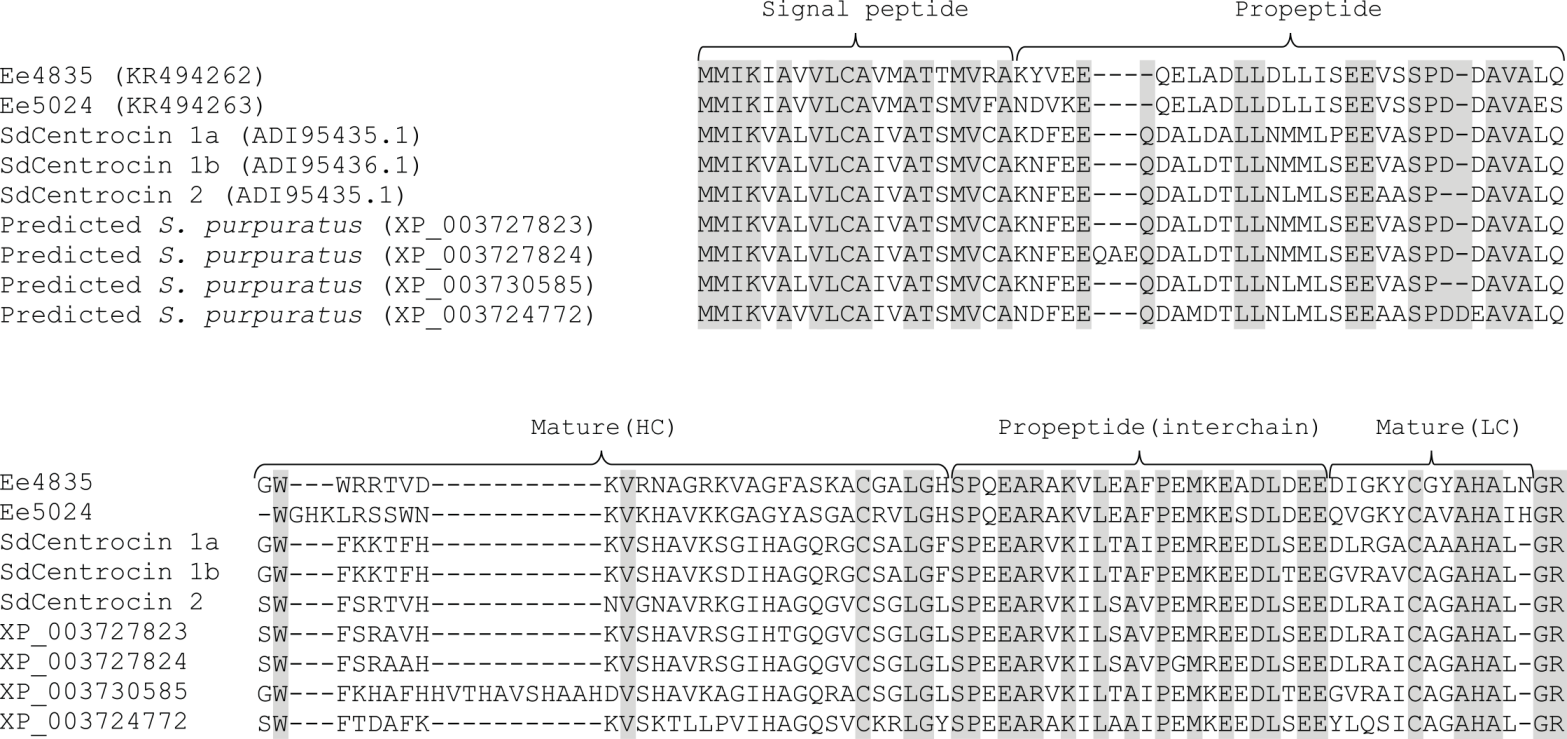
Multiple sequence alignment of Ee4835 and Ee5024 with homologues in *S*. *droebachiensis* and *S*. *purpuratus*. In the aligned sequences, grey indicates identical amino acids. The predicted signal peptides, propeptides and mature peptides are marked with curly brackets. Gaps are inserted to maximise similarity. In the top row, accession numbers are given in parentheses, the mature peptide sequences are presented in the bottom rows.

**Fig 4:**
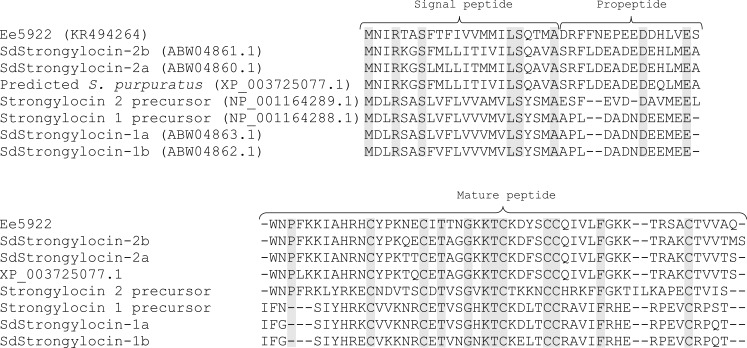
Multiple sequence alignments of Ee5922 with homologues in *S*. *droebachiensis* and *S*. *purpuratus*. In the aligned sequences, grey indicates identical amino acids. The predicted signal peptides, propeptides and mature peptides are marked with curly brackets. Gaps are inserted to maximise similarity. In the top row accession numbers are given in parentheses, the mature peptide sequences are presented in the bottom rows.

To elucidate the complete peptide sequences, degenerated primers were designed according to the partial primary peptide sequences. The constructed 3’ RACE-Ready cDNA library was employed as template to amplify the 3’-end of the transcripts. Three partial cDNA clones of ~320 bp, ~400 bp and ~370 bp were cloned and sequenced. These encoded the C-terminal end of the purified peptides Ee4835, Ee5024 and Ee5922 respectively. No cDNA sequence matching Ee4635 was found. Using the 5’RACE-PCR approach, 5’ end cDNA sequences of Ee4835, Ee5024 and Ee5922 were cloned. The cDNA of Ee4835 was 660 bp in length with an open reading frame of 360 bp encoding a polypeptide of 119 amino acids (Fig 3). The theoretical *pI* and MW of the Ee4835 precursor were calculated to be 5.18 and 13040.0 Da respectively. The cDNA of Ee5024 was 676 bp in length with an open reading frame of 366 bp encoding a polypeptide of 121 amino acids (Fig 3). The theoretical *pI* and MW of the Ee5024 precursor were calculated to be 5.69 and 13151.1 Da respectively. The cDNA of Ee5922 was 675 bp in length with an open reading frame of 267 bp encoding a polypeptide of 89 amino acids (Fig 4). The theoretical *pI* and MW of the Ee5922 precursor was calculated to be 8.58 and 10297.96 Da respectively.

Based on the deduced amino acids from cDNA analysis, the non-identified amino acids during Edman degradation sequenced in the two peptides Ee4835 and Ee5024 were found to be Trp residues. The N-terminal amino acid in the peptide Ee5922 was also shown to be Trp. Since Trp is normally detected during Edman degradation, these Trp-residues are likely modified. The precursor molecules of the peptides Ee4835 and Ee5024 have preprosequences. Analysis with SignalP 4.1 using the neural network model with SignalP-noTM setting showed that the highest calculated cutoff value was located between positions 20 and 21 in the N-terminal sequence of both proteins. Therefore, the predicted signal peptides consist of the 20 N-terminal amino acids followed by a prosequence of 30 amino acids (Fig 3). The native peptides with a proposed dimeric structure start at Gly-51 in Ee4835 and Trp-51 in Ee5024. A 24 amino acid interchain sequence separates the HCs from the LCs and is not present in the mature peptides. The two interchains (belonging to Ee4835 and Ee5024) are very similar, differing in only one amino acid residue. The LCs of both peptides seem to consist of 15 amino acids and both peptides contain a C-terminal dipeptide (Gly-Arg) which, based on Edman degradation and MW data, is cleaved of. The theoretical *pI* of the deduced mature peptides was 10.04 for both, indicating a cationic character. *In silico* analysis of the peptide Ee5922 suggested that the first 22 amino acids represent a signal peptide followed by a prosequence containing 16 amino acids (Fig 4). The native, cysteine-rich peptide starts at Trp-39.

All peptides discovered in the present study have an abundance of positively charged amino acids (23–29%) and hydrophobic amino acids (29–42%). This indicates that these sequences are cationic and have the possibility to form an amphipathic structure, a feature which is common for most AMPs and considered important for their antimicrobial activity [2, 44–48]. Their positive charge will aid in the electrostatic attraction between the peptide and the anionic microbial membranes [49–52]. Distributing positively charged residues on one side and hydrophobic residues on the other side of the structure (i.e. amphipathic), allows the peptide to incorporate itself into and act on the bacterial membranes, as suggested by several authors [2, 45, 52]. All three peptides contain Trp residues which are considered especially important for peptide hydrophobicity and the interactions in the membrane-water interface [48].

### 3.3 Homology searches and bioinformatics

BLAST searches performed on the deduced full-length amino acid sequences of Ee4835, Ee5024 (Fig 3) and Ee5922 (Fig 4) revealed homology to the centrocins and strongylocins isolated from *S*. *droebachiensis* [14, 23]. Several putative proteins in *S*. *purpuratus* were also found to be homologues of the Ee-peptides. Due to their apparent similarity with centrocins and strongylocins (strongylocin 2 primarily), the names EeCentrocin 1 (Ee4835) and 2 (Ee5024) and EeStrongylocin 2 (Ee5922) are proposed for the peptides characterised in the present study. For clarity, the originally characterised centrocins and strongylocins (from *S*. *droebachiensis*) will be referred to as SdCentrocins and SdStrongylocins for the remainder of this paper.

Alignment of EeCentrocins, SdCentrocins and predicted homologues in *S*. *purpuratus* displays high similarity in the preprosequence region (58–67%) and in the interchain regions (58–79%), but not in the HC or LC regions (Fig 3) which display a much greater individual diversity. This suggests that the HCs and LCs are subject to a much higher mutation rate and that the signal sequences and pro sequences are more conserved regions. The partial peptide sequence of Ee4635 did not return any homologous sequences using NCBI BLAST search analysis. However, analysis performed by the built-in BLAST search engine of the LAMP database revealed low (E-value of 0.59) but significant homology to SdCentrocin 1. Based on the fact that the sequence also contains one cysteine residue, unidentified or modified residues and an abundance of positively charged and hydrophobic amino acids, it could be hypothesised that this peptide too belongs to the diverse heavy chains of the centrocins.

*In silico* analysis of the complete EeCentrocin sequences indicates that the first 20 amino acids in the precursors function as signal peptides. Furthermore, the data show that the precursor molecules have a first prosequence region of 30 amino acids, followed by a heavy chain sequence (30 and 32 amino acids for EeCentrocin 1 and 2 respectively), a second prosequence region of 24 amino acids (interchain), a light chain sequence of 15 amino acids and finally a prosequence consisting of two amino acids. The function of these prosequences is unknown, but they might aid in proper folding of the active peptides and/or function as a target for site-specific proteases [23, 53].

Alignment of EeStrongylocin 2, SdStrongylocins and predicted homologues in *S*. *purpuratus* (Fig 4) displays similarities in the presequence region (50–59%), the prosequence region (12–50%) and in the mature peptide (40–84%). EeStrongylocin 2 displays greatest identity with SdStrongylocin 2a and 2b (68–69%). *In silico* analysis defined the first 22 N-terminal amino acids as the signal peptide, leaving a 16 amino acid prosequence before the mature peptide. As centrocin-like and strongylocin-like peptide sequences have been discovered in three species of sea urchins, the possibility of them being a trait of this class of echinoderms exist, increasing the possibility of discovering other homologous bioactive peptides in other species of sea urchins.

**Fig 5:**
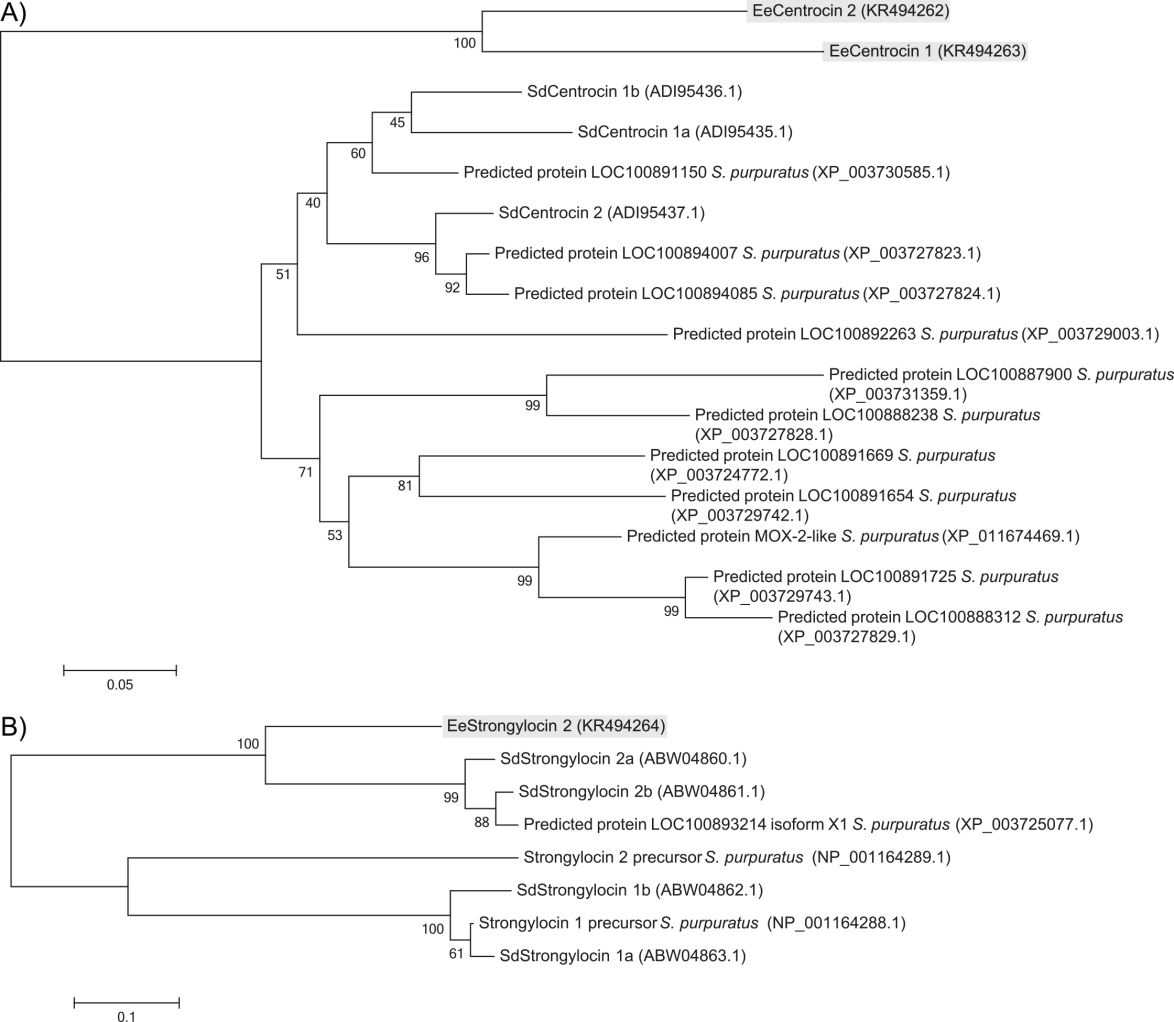
**Evolutionary relationships of A) centrocins and B) strongylocins identified in *E*. *esculentus*, *S*. *droebachiensis* and *S*. *purpuratus*.** The evolutionary history was inferred using the Neighbour-joining method [39] and the optimal trees are shown. The percentage of replicate trees in which the proteins clustered together during the bootstrap test (500 replicates) is given next to the nodes [55]. The tree is drawn to scale, with branch lengths in the same units as those of the evolutionary distances used to infer the phylogenetic tree. The evolutionary distances were computed using the Poisson correction method [40]. Accession numbers are given in parentheses.

Fig 5 represents the phylogenetic trees composed of all homologues found in *S*. *droebachiensis* and *S*. *purpuratus* by BLAST searches. It appears that the EeCentrocins (Fig 5A) are phylogenetically separated from the other peptides, sharing one common ancestor with all. The SdCentrocins are more closely related to the predicted centrocin-like proteins in *S*. *purpuratus*. The SdStrongylocin 1 and 2 AMPs seem to be separated phylogenetically (Fig 5B) but share a common ancestor. EeStrongylocin 2 aligns more closely with SdStrongylocin 2. The *S*. *purpuratus* genome has been completely sequenced [54] whereas only a few proteins from *S*. *droebachiensis* and *E*. *esculentus* have been sequenced. It is therefore unknown whether the two species *E*. *esculentus* and *S*. *droebachiensis* contain additional strongylocin-like and centrocin-like peptides or not, and explains the excess of sequences from *S*. *purpuratus* in the figures.

### 3.4 Characterisation of post-translational modifications

**Fig 6:**
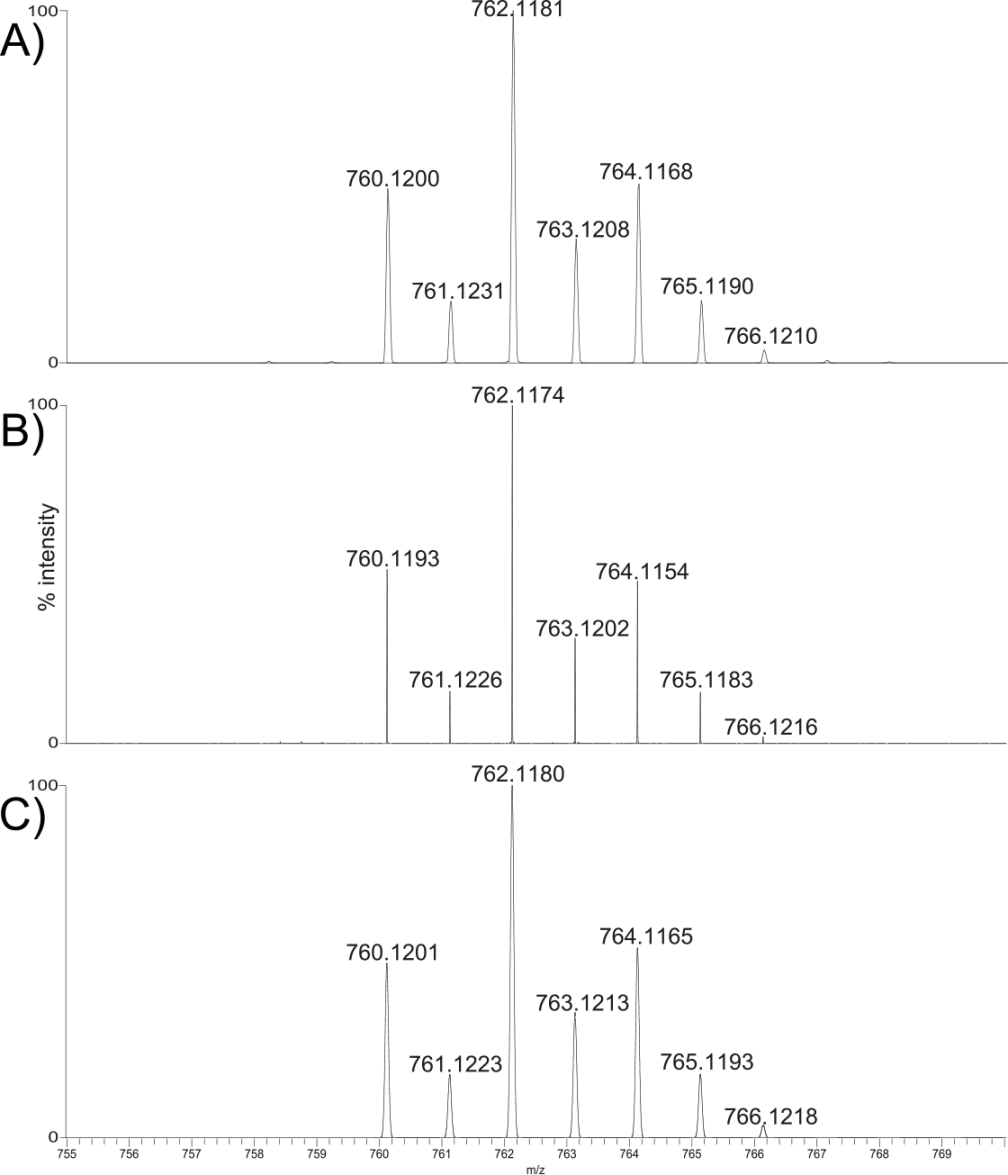
Characterisation of the post-translational modifications of EeCentrocin 1 HC. High-resolution mass spectra showing the *m/z* isotope cluster corresponding to the [M+H]^+^ ions of A) Main peptide fragment from a tryptic digest of native EeCentrocin 1, B) Synthesised fragment GW_Br_W_Br_R, and C) Calculated isotope distribution of [GW_Br_W_Br_R+H]^+^. The identical and distinctive distributions (A, B and C) of the singly charged isotopes indicate the presence of two Br-Trp residues in EeCentrocin 1.

The theoretical monoisotopic mass of EeCentrocin 1 (deduced from cDNA) containing an intramolecular disulphide bond was calculated to be 4675.37 Da, leaving a gap of 154.83 Da to the native isolated peptide (4830.20 Da). In order to identify the modifications of the second and third amino acids (both being Trp residues according to cDNA), the peptide was degraded by trypsin and analysed by HR-MS. The mass value of the major fragment and its corresponding isotope distribution of [M+H]^+^ ions (Fig 6A) agreed well with the ion distribution of the synthetically produced fragment GW_Br_W_Br_R (Fig 6B) and a theoretical ion distribution of [GW_Br_W_Br_R+H]^+^ (Fig 6C), indicated that the peptide contains two brominated Trp residues. Seven ion peaks are clearly visible in all three figures with similar relative abundances and minute differences in *m/z* values (See S7 Table for calculated and measured *m/z* values). The presence of two Br-Trp in EeCentrocin 1 leads to a theoretical monoisotopic mass of 4831.19 Da. This exceeds the measured mass by approximately one Da, suggesting that one of the peptide chains is amidated at the C-terminal (4830.20 Da).

The deduced sequence of the EeCentrocin 1 LC holds a dipeptide “Gly-Arg” at the C-terminal end, which resembles previously published amidation signal sequences. As shown by HR-MS (S3 Fig), the LC of the isolated peptide is in fact amidated at the C-terminal. The tachyplesin precursor from the horseshoe crab (*Tachypleus tridentatus*) [56], aureins (excluding aurein 5.3) from the frog (*Litoria aurea*) [57] and astacidin 2 from the fresh water crayfish (*Pacifastacus leniusculus*) [58] all contain amidation signals such as “Gly-Lys” and “Gly-Lys-Arg” which leads to a C-terminal amidation. However in the centrocins [23] of *S*. *droebachiensis*, no amidation is observed despite the presence of the cleaved-off “Gly-Arg” C-terminally.

No MS data fits the theoretical monoisotopic mass (4881.56 Da) calculated from the deduced amino acid sequence of EeCentrocin 2. Based on the findings from EeCentrocin 1 and the bromination occurring in centrocins [23], we hypothesise that EeCentrocin 2 also has two post-translationally brominated Trp residues (positions 1 and 9) in the HC. This is also based on the inability of Edman degradation to identify any residue in these positions. Adding two bromines to the elemental composition leads to a theoretical mass of 5037.38. This deviates from the isolated peptide by +18.00 Da, indicating the presence of additional modifications on the isolated AMP. The deduced LC sequence of EeCentrocin 2 also contains a “Gly-Arg” amidation signal, which indicates that the peptide contains an amidated His residue C-terminally. Additionally, the LC contains an N-terminal Gln residue. The formation of pyroglutamic acid (Glp) is known to occur both enzymatically and spontaneously [59, 60] when Gln or Glu is located N-terminally in a peptide sequence. HR-MS and MS/MS data of the alkylated LC of EeCentrocin 2 (S4 Fig) supports formation of both N-terminal Glp and C-terminal amidation, leading to a theoretical monoisotopic mass of 5019.37 Da, which corresponds to the measured mass. Glp-formation also explains why no sequence for the LC was obtained during Edman degradation as it sequences α-amino groups [61]. In The Antimicrobial Peptide Database, there are 19 entries with N-terminal Glp of which one is of marine origin [62]. The measured monoisotopic mass of EeStrongylocin 2 corresponds to the amino acid sequence deduced from cDNA. The theoretical MW of the peptide with 3 disulphide bonds is 5839.86 Da, and by replacing an indole hydrogen with a bromine, the theoretical monoisotopic mass (5917.80 Da) matches the experimental one (5917.77 Da). Additionally, a brominated ion at *m/z* 769.19 emerged during LC-PDA-MS with the same retention time as EeStrongylocin 2 that probably represent H-W(Br)NPFK-OH (S5 Fig).

**Fig 7:**
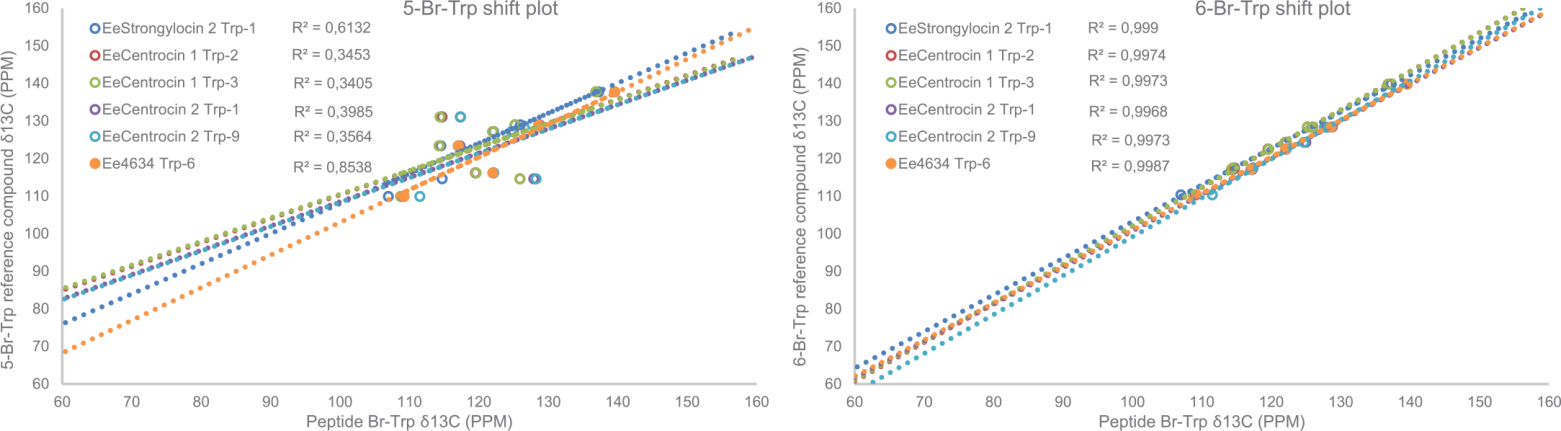
Carbon chemical shifts of Br-Trp in native peptides correlated with synthetic 5-Br-Trp and 6-Br-Trp. The chemical shifts are less well correlated for the 5-Br reference compound (left, 5.36 ppm average error) than those of the 6-Br reference compound (right, 1.55 ppm average error). Each data point in the figure represents a carbon of the indole of Trp.

NMR spectroscopy was employed to characterise the position of the bromine on the Trp residues of the native peptides, including the two-peptide mixture of EeCentrocin 2 and Ee4635. The native proton resonances of the brominated Trp residues of EeCentrocin 1 and EeStrongylocin 2 were compared to those of the reference compounds 4-, 5-, 6-, and 7-Br-Trp in water (S6 Fig). The proton coupling patterns did not match the patterns of 4- and 7-Br-Trp, leaving 5- and 6-Br-Trp as the only viable alternatives. The chemical shifts of the peptides are found to slightly better match with the 6-Br reference compound (average error: 0.14 ± 0.07 ppm) in comparison with 5-Br (average error: 0.16 ± 0.12 ppm). Well-resolved aromatic region ^1^H,^13^C-HSQC and ^1^H,^13^C-HMBC of the modified Trp residues of EeCentrocin 1 and EeStrongylocin 2 revealed the positions of the brominations (S7 Fig). The carbon chemical shifts matched far better with reference compounds of 6-Br-Trp (Fig 7 and S7 Fig). This led to the confirmation of, not only 6-Br-Trp in EeCentrocin 1 and EeStrongylocin 2, but also two 6-Br-Trp residues in EeCentrocin 2 where only cDNA data (displaying Trp) existed for positions 1 and 9. One other 6-Br-Trp residue was identified, likely to belong to the 6^th^ amino acid in Ee4635 as the chemical shifts of a 6-Br-Trp in the first position of Ee4635 would perfectly overlap with EeCentrocin 2 due to identical N-terminals.

Marine species are well known to incorporate bromine in their secondary metabolites and peptides [63, 64]. Bromination of Trp in the 6 position as a post-translational modification was first reported in 1997 in toxins isolated from cone snails *Conus imperialis* and *C*. *radiatus* [63]. Styelin D from the marine tunicate (*Styela clava*) also has a confirmed 6-bromination of Trp [65]. Several other marine organisms have brominated Trp residues but without confirmed positioning: cathelicidins from the Atlantic hagfish (*Myxine glutinosa*) [49], hedistin from the marine annelid (*Nereis diversicolor*) [66], strongylocin 2 and the centrocins from the green sea urchin (*S*. *droebachiensis*) [14, 23]. The biological function of bromine-substitution of Trp is not known, but has been suggested to aid in the proteolytic protection of the peptides [49].

**Fig 8:**
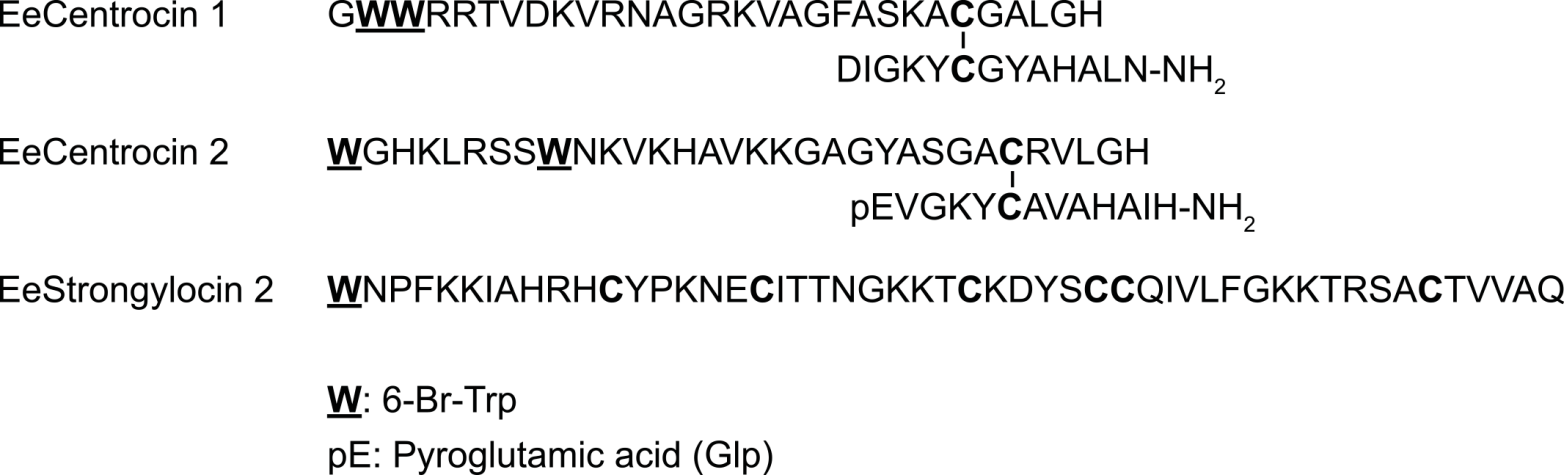
Structures of peptides discovered in *E*. *esculentus*. The two top structures are the heterodimeric EeCentrocins 1 and 2. The disulphide bonds connecting the HC and LC are indicated with “|”. The lower sequence is the primary structure of EeStrongylocin 2. All Trp substitutions are assigned to the 6 position of the indole.

No ordered structure of the centrocin-peptides in MQ-H_2_O and with high salt concentrations has been detected during the NMR experiments. However, several peptides only adopt an ordered structure in the presence of membranes or membrane-mimics [52]. The secondary structure of EeStrongylocin 2 as dictated by its disulphide bonds is yet to be elucidated. The proposed structure of the two dimeric peptides EeCentrocins 1 and 2, and the primary structure of EeStrongylocin 2 can be viewed in Fig 8.

### 3.5 Bioactivities

The antimicrobial activity of the native EeCentrocin 1 was measured against four selected bacterial strains (Table 2, raw data in S9 Table). The peptide displayed potent antibacterial activity against the Gram-positive bacteria, *C*. *glutamicum* and *S*. *aureus* (MIC = 0.78 μM against both) and the Gram-negative bacteria, *E*. *coli* and *P*. *aeruginosa* (MIC = 0.1 and 0.78 μM respectively). The activity was in the same range as for the SdCentrocins [23]. Because of a challenging purification, we were not able to perform investigations of the antibacterial potency of the native EeCentrocin 2 and Ee4635. However, the mix of both these peptides was antibacterial. The antibacterial potency fluctuates enormously among amphipathic AMPs [67–69], but EeCentrocin 1 (and the HC of EeCentrocins 1 and 2) are antimicrobial in similar ranges as those of the SdCentrocins [23]. According to previous published work [51], this is a typical activity range for AMPs. It has also been stated that the MIC activity range of an AMP rarely falls below 0.5–1.0 μM [50], which is in the same MIC region as the most potent AMPs presented in this paper.

**Table 2:** Antimicrobial activities of native EeCentrocin 1, EeStrongylocin 2 and synthesised analogues of EeCentrocin 1 and 2. LC = light chain, HC = heavy chain, HC-diBr = peptide with two brominated Trp residues.

	Minimum inhibitory concentration (MIC) (μM)					
	EeCentrocin 1				EeCentrocin 2	EeStrongylocin 2
Microorganism	Native	HC-diBr	HC	LC	HC	Native
Gram-positive bacteria						
*B*. *subtilis*	nt^1^	1.56	1.56	>100	6.25	nt
*C*. *glutamicum*	0.78	0.78	0.39	>100	0.78	1.56
*S*. *aureus*	0.78	3.13	3.13	>100	6.25	3.13
*S*. *epidermidis #*1	nt	6.25	6.25	>100	3.13	nt
*S*. *epidermidis* #2	nt	3.13	3.13	>100	3.13	nt
Gram-negative bacteria						
*E*. *coli* #1	0.1	1.56	0.78	>100	1.56	0.78
*E*. *coli* #2	nt	1.56	1.56	>100	6.25	nt
*P*. *aeruginosa* #1	0.78	1.56	1.56	>100	0.78	1.56
*P*. *aeruginosa* #2	nt	3.13	3.13	>100	6.25	nt
Fungi						
*A*. *pullulans*	nt	6.25	12.5	>100	25	nt
*C*. *albicans*	nt	50	100	>100	50	nt
*Cladosporium sp*.	nt	>100	>100	>100	12.5	nt
*Rhodotorula sp*.	nt	12.5	12.5	>100	3.13	nt
*S*. *cerevisiae*	nt	>100	50	>100	12.5	nt

nt: not tested

The HC of the EeCentrocins was shown to be the antimicrobial portion of the peptides, displaying similar potency as the native peptide against some bacterial strains (Table 2). Interestingly, the activity of the HC also appears to be independent of the bromination of the Trp residues. The MICs of EeCentrocin 1 HC-diBr towards bacteria ranged from 0.78 to 6.25 μM which is almost identical to the MIC displayed by EeCentrocin 1 HC (0.39–6.25 μM). The HC of EeCentrocin 2 displayed similar antibacterial activities (MIC ranging from 0.78–6.25 μM). The antifungal activities of the two EeCentrocin 1 HCs were also quite similar, differing only by one dilution step, whereas EeCentrocin 2 HC seems to be a slightly more potent antifungal agent. The LC (synthesised with a C-terminal carboxyl group) of EeCentrocin 1 is of no observable importance to antimicrobial activity, when comparing the native peptide with the dibrominated or nonbrominated HCs, and it is not antimicrobial when tested alone. This supports our previous studies on the LC of the SdCentrocins [23] and suggests other tasks for the LC. Whether a C-terminally amidated peptide (increasing the charge by +1) would have displayed antimicrobial activity is uncertain.

The MIC of native EeStrongylocin 2 was found to range from 0.78 to 3.13 μM against the tested strains. The SdStrongylocins displayed similar antibacterial activity with MICs ranging from 2.5 to 5 μM [14]. The two recombinantly produced peptides, SpStrongylocins 1 and 2 (originating from *S*. *purpuratus*) displayed MICs towards the same strains at 15 and 7.5 μM respectively [24].

**Fig 9:**
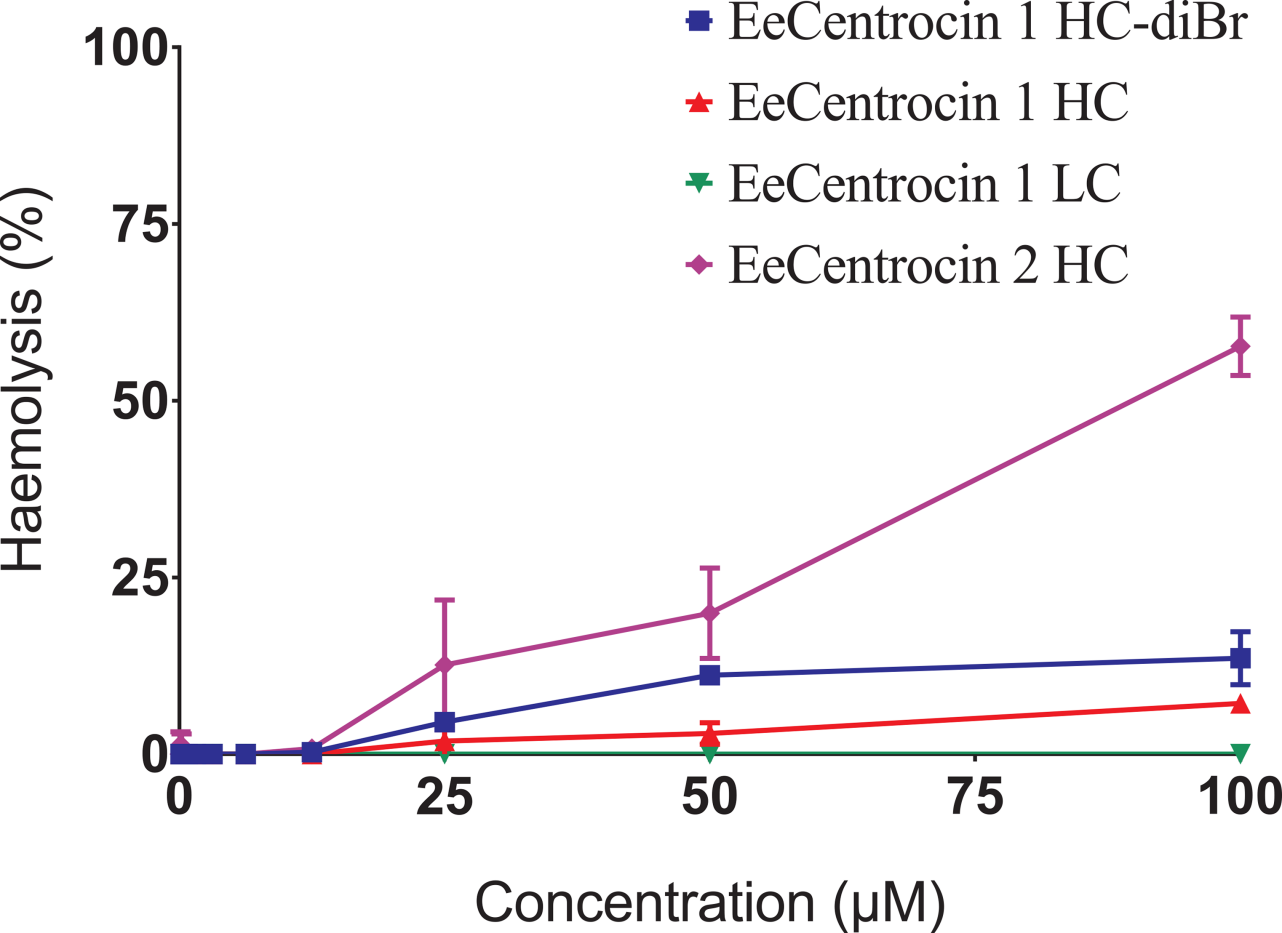
Haemolytic activity of synthetic analogues of EeCentrocin 1 and 2. The different synthetic peptide analogues of EeCentrocin 1 and 2 display minor haemolytic activity in concentrations up to 12.5 μM. EeCentrocin 2 HC displays the highest haemolytic activity (56.4% haemolysis at 100 μM).

The synthesised EeCentrocin 1 (HC, HC-diBr and LC) peptide analogues displayed no or minor haemolytic activity at a concentration of 100 μM (Fig 9, raw data in S10 Table), a concentration which is 16 times higher than the MIC against the least sensitive bacteria (Staphylococci) tested in this study. No or minor haemolytic activity is a prerequisite if the peptides are ever to be exploited clinically or as food additives [70]. EeCentrocin 2 HC was more haemolytic, displaying 11.7, 18.9 and 56.3% haemolysis at concentrations 25.0, 50.0 and 100 μM, respectively. The reason for this higher haemolytic activity of EeCentrocin 2 compared to EeCentrocin 1 is unclear. However, increasing physicochemical parameters like hydrophobicity and hydrophobic face are known to enhance the haemolytic activity of α-helical AMPs [71].

## 4 Conclusion

The widespread use of antibiotics and the associated development of microbial resistance to these drugs has emerged as a major global problem. The search for new antibacterial agents has therefore become an important area in natural product drug discovery. Cationic AMPs have previously been isolated from various species and phyla, and represent a novel class of antibiotics. In this study, potent AMPs were for the first time characterised from coelomocyte extracts of the Edible sea urchin, *E*. *esculentus*, collected from sub-Arctic waters. Three 5–6 kDa AMPs were shown to be novel members of the centrocin and strongylocin families of AMPs. The EeCentrocins have a heterodimeric structure composed of a heavy chain and a light chain connected by a single disulphide bond. Neither the presence of brominated amino acids in the heavy chain nor a light chain with C-terminal amidation and N-terminal pyroglutamic acid seem to be necessary for maintaining antibacterial activity, but these additional structures might aid in the protection against proteolytic degradation of the peptides. The secondary structure and the three-dimensional conformation of EeStrongylocin 2 as dictated by its three disulphide bonds remains unknown, but should be explored. Future research should also include mode of action studies, where cellular targets are identified, and structure-activity relationship studies where truncated analogues of the EeCentrocin HCs are constructed in order to pinpoint the pharmacophore. Additionally, as it appears that sea urchins in general are producers of strongylocins and centrocins, a genomic approach to discover homologues in other sea urchins (or echinoderms) for the discovery of novel AMPs could be a beneficial venture.

This study has demonstrated that marine invertebrates are a valuable resource for discovering unique bioactive peptides, providing promising leads for development of novel antimicrobial drugs.

## Supporting Information

S1 Fig **ESI-MS spectra of A) native Ee4635 (M1) and Ee5024 (M2), B) native Ee5922 and C) native Ee4835.** The peptide Ee4635 revealed multiple ions at *m/z* 773.61 [M_1_ + 6H]^6+^, 927.95 [M_1_ + 5H]^5+^, 1159.65 [M_1_ + 4H]^4+^, and 1545.93 [M_1_ + 3H]^3+^, indicating that the peptide has a MW of approximately 4635 Da. The peptide Ee5024 revealed multiple ions at *m/z* 838.30 [M_2_ + 6H]^6+^, 1005.92 [M_2_ + 5H]^5+^, 1257.06 [M_2_ + 4H]^4+^, and 1675.61 [M_2_ + 3H]^3+^, indicating that the peptide has a MW of approximately 5024 Da. ESI-MS of Ee5922 revealed multiple ions at m/z 741.37 [M + 8H]^8+^, 847.03 [M + 7H]^7+^, 988.01 [M + 6H]^6+^, 1185.47 [M + 5H]^5+^, 1481.69 [M + 4H]^4+^, and 1975.09 [M + 3H]^3+^, indicating that the peptide has a MW of approximately 5922 Da. ESI-MS of Ee4835 revealed multiple ions at m/z 691.73 [M + 7H]^7+^, 806.74 [M + 6H]^6+^, 968.01 [M + 5H]^5+^, 1209.68 [M + 4H]^4+^, and 1612.72 [M + 3H]^3+^, indicating that the peptide has a MW of approximately 4835 Da.
(EPS)

S2 Fig **HR-MS spectra of the [M+5H]^5+^ ions of A) Ee4635, B) Ee5024, C) Ee4835 and the [M+6H]^6+^ ion of D) Ee5922**.
(EPS)

S3 Fig HR-MS spectrum of the [M+2H]^2+^ ion of the alkylated (4-vinylpyridine) LC of EeCentrocin 1. The measured *m/z* (764.87) corresponds with the LC containing a C-terminal amidation (theoretical *m/z* 764.87).
(EPS)

S4 Fig **HR-MS of the alkylated (4-vinylpyridine) EeCentrocin 2 LC A) [M+H]**^**+**^ **ion, and B) MS/MS spectrum of the expected a2 and b2 ions.** The measured *m/z* (1483.76) corresponds with the LC containing a C-terminal amidation and an N-terminal Glp (theoretical *m/z* 1483.77). The MS/MS spectrum supports the formation of Glp with the a2 ion at *m/z* 183.11 and b2 ion at *m/z* 211.11.
(EPS)

S5 Fig ESI-MS spectrum of a brominated ion possibly representing the N-terminal sequence H-W(Br)NPFK-OH of EeStrongylocin 2. The fragment appeared in the same MS spectrum as EeStrongylocin 2 during LC-PDA-MS. The measured *m/z* (769.2) on low-resolution MS corresponds with the theoretical *m/z* (769.3).
(EPS)

S6 Fig NMR assignment of the 6-Br-Trp residues of native EeCentrocin 1, EeStrongylocin 2 and brominated reference compounds. A-D) ^1^H spectra of the reference compounds 4, 5, 6 and 7-Br-Trp, E) native EeCentrocin 1 and F) EeStrongylocin 2 with putative assignments for the two Br-Trp residues compared to the reference compounds positions.
(EPS)

S7 Fig 6-Br-Trp carbon assignments in superimposed HSQC and HMBC spectra. A) Reference compound 6-Br-Trp, B) Reference compound 5-Br-Trp, C) EeCentrocin 1, D) EeCentrocin 2, E) Ee4635 and F) EeStrongylocin 2.
(EPS)

S1 Table Antibacterial activity of SPE eluates and the organic phase of *E*. *esculentus* coelomocytes against Gram-positive and Gram-negative bacteria. (XLSX)

S2 Table MS experiment and parameters. The experiments used for mass spectrometry are listed for low resolution above and high resolution below.
(XLSX)

S3 Table Overview of primers used in this study. (XLSX)

S4 Table NMR experiments and acquisition parameters for synthetic EeCentrocin 1 HC-diBr collected in H_2_O:D_2_O 9:1. (XLSX)

S5 Table NMR experiments and acquisition parameters for the native EeCentrocin 1 collected in D_2_O. (XLSX)

S6 Table NMR experiments and acquisition parameters for the native Ee4635/EeCentrocin 2 mix and EeStrongylocin 2 collected in D_2_O. (XLSX)

S7 Table Predicted and measured *m/z* values of the GW(Br)W(Br)R fragment of EeCentrocin 1. (XLSX)

S8 Table Backbone and side chain assignment of synthetic EeCentrocin 1 HC-diBr. The table depicts chemical shifts of the synthetic HC-diBr.
(XLSX)

S9 Table Raw data from antimicrobial activity testing. Grey highlights are the values exported to the table displayed in the manuscript.
(XLSX)

S10 Table Raw data from haemolytic assay showing absorbance at 550 nm. Mean values are exported to the figure used in the manuscript.
(XLSX)
